# The London Medical Directory, 1845. Containing the Name, Address, Qualification, Official Appointments, Honorary Distinctions, and Literary Productions of Every Physician, Surgeon, and General Practitioner Resident in London

**Published:** 1845-07

**Authors:** 


ART. IX.—1. *The London Medical Directory,* 1845. *Containing the name, address, qualification, official appointments, honorary distinctions, and literary productions of every Physician, Surgeon, and General Practitioner resident in London.—London,* 1845. 8vo, pp. 180.

2. *The Medical Directory of Great Britain and Ireland, for* 1845.—*London,* 1845. 8vo, pp. 672.

THE title-page of the first of these volumes, which we have transcribed in full, gives a correct representation of its object and substance. As far as we can judge from occasional inspection of its contents, when needing its assistance, we think the information contained in it both extensive and accurate. As a record of the literary labours of metropolitan practitioners, it cannot fail to be both useful and interesting; and as the first attempt made in this country to obtain and make public this sort of knowledge, it may justly claim the attention and patronage of the profession. In future editions it will, doubtless, be both enlarged and improved.

The second work is on a much more extended scale, and has been compiled on a different plan and with different views. It has no pretension beyond that of being a mere directory of names and addresses; but as it professes to record those of the whole profession throughout the three kingdoms, its compilation must have been a work of immense labour. It contains three several Directories, one for England and Wales, one for Scotland, and one for Ireland; and gives, besides, lists of all the poor-law unions in England, with the surgeons' names; of the lunatic asylums; of the medical officers of the army and navy, &c. The Directory of England contains—1, a list of all medical men qualified to practise in England and Wales; 2, a list of all the medical men practising in London under two heads, physicians and surgeons; 3, a list of all the towns in England, with the names of the medical men in each.

Such a work as this cannot fail to be useful if carefully compiled. As might perhaps be expected in a first essay, the volume contains a vast number of errors and imperfections, which it is to be hoped may be corrected and removed in subsequent editions.

